# Effects of poly(L-lactide-ε-caprolactone) and magnesium hydroxide additives on physico-mechanical properties and degradation of poly(L-lactic acid)

**DOI:** 10.1186/s40824-016-0054-6

**Published:** 2016-03-15

**Authors:** Eun Young Kang, Eugene Lih, Ik Hwan Kim, Yoon Ki Joung, Dong Keun Han

**Affiliations:** Center for Biomaterials, Korea Institute of Science and Technology, Hwarangno 14-gil 5, Seongbuk-gu, Seoul 02792 South Korea; Department of Biological Science, Korea University, Anam-ro 145, Seongbuk-gu, Seoul 02841 South Korea; Department of Biomedical Engineering, Korea University of Science and Technology, Gajeong-ro 217, Yuseong-gu, Daejeon 34113 South Korea

**Keywords:** Poly(L-lactic acid), Poly(L-lactide-ε-caprolactone), Magnesium hydroxide, Thermal decomposition, Neutralization

## Abstract

**Background:**

Biodegradable poly(L-lactic acid) (PLLA) is one of the most widely used polymer in biomedical devices, but it still has limitations such as inherent brittleness and acidic degradation products. In this work, PLLA blends with poly(L-lactide-ε-caprolactone) (PLCL) and Mg(OH)_2_ were prepared by the thermal processing to improve their physico-mechanical and thermal properties. In addition, the neutralizing effect of Mg(OH)_2_ was evaluated by degradation study.

**Results:**

The elongation of PLLA remarkably increased from 3 to 164.4 % and the glass transition temperature (T_g_) of PLLA was slightly reduced from 61 to 52 °C by adding PLCL additive. Mg(OH)_2_ in polymeric matrix not only improved the molecular weight reduction and mechanical strength of PLLA, but also neutralized the acidic byproducts generated during polyester degradation.

**Conclusions:**

Therefore, the results demonstrated that the presence of PLCL and Mg(OH)_2_ additives in PLLA matrix could prevent the thermal decomposition and control degradation behavior of polyester.

## Background

**Fig. 1:**
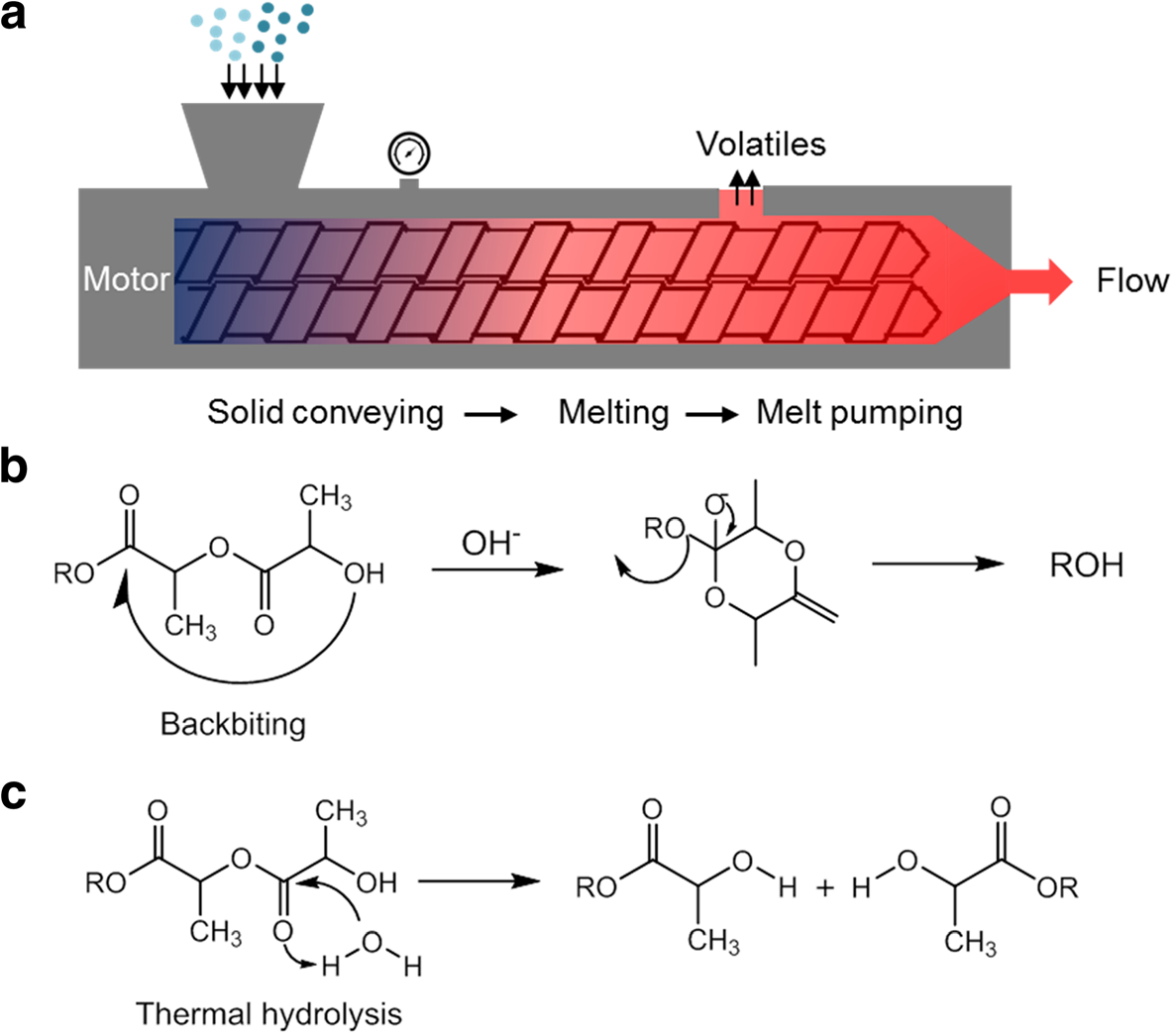
**a** Thermal extrusion process and **b**, **c** degradation mechanisms of PLLA

Poly(L-lactic acid) (PLLA) has excellent mechanical properties, thermal plasticity, and transparency, which is a renewably derived thermoplastic and biodegradable polyester extensively investigated over the last several decades [1–3]. However, a few drawbacks of PLLA including inherent brittleness, poor melt strength, and imperfect biocompatibility have limited numerous applications in biomedical science and engineering such as implant devices, tissue scaffolds, and internal sutures [4, 5]. In particular, thermal extrusion process at high temperature (Fig. 1a), which is necessary to manufacture various biodegradable devices based on PLLA, maximizes inherent flaws of PLLA such as poor mechanical properties, matrix cracking, and thermal instability *via* random chain scission [6], depolymerisation [7] and intramolecular transesterification [7, 8]. Fig. 1b and c show the degradation mechanism of PLLA based on backbiting and hydrolytic degradations. The acidic byproducts generated in this reaction acted as a nucleophile in ester linkage and accelerated the degradation reaction of PLLA backbone. Moreover, the presence of moisture in PLLA accelerates the cleavage of the ester linkage with the acid and alcohol groups that induced a significant molecular weight reduction. In biomedical applications, the released acidic degradation products from PLLA matrix reduce the pH of implant environment and result in inflammatory reaction in the body [9].

One of the most important disadvantages of PLLA is brittleness due to its semi-crystalline character, which can limit its applicability. Many papers have reported the PLLA blends with ductile and biodegradable polymers such as polyglycolic acid (PGA) [10, 11], polyamide 6 (PA6) [12] and poly(butylene succinate-co-adipate) (PBSA) [13, 14]. Aliphatic polyester based on polycaprolactone such as poly(L-lactide-ε-caprolactone) (PLCL) has been used to improve the processability and thermal stability of PLLA because PLCL has flexible characteristics, low melting temperature, and aliphatic chain of polycaprolactone segment. The PLLA blends with polycaprolactone based polyester exhibit remarkable increase in their elongation at break due to relative increase of the amorphous region compared with PLLA [15–17].

Magnesium hydroxide, which is widely used as an antacid agent, is a basic inorganic compound with the chemical formula of hydrated Mg(OH)_2_. Mg(OH)_2_ is *in vivo* absorbable in the form of magnesium and hydroxide ions as well as biocompatible [18]. The magnesium ions can be interact with anionic compounds and neutralize the acidic degradation products of PLLA [19]. In addition, Mg(OH)_2_ also can be used to mechanically reinforce the PLLA matrix, which is a key factor resulting in effective improvement in mechanical properties [20].

In this work, the PLLA blends with PLCL and Mg(OH)_2_ additives were developed by thermal processing to improve the flexibility and thermal stability of PLLA matrix and enhance the tensile strength of thermally-processed PLLA. The physic-mechanical properties of PLLA control and PLLA/PLCL blends were measured by gel permeation chromatography (GPC) and universal testing machine (UTM), and their thermal properties were evaluated by using differential scanning calorimetry (DSC) and thermogravimetric analysis (TGA). The effect of Mg(OH)_2_ in PLLA and PLLA/PLCL matrix was determined by measuring molecular weight and mechanical properties such as tensile strength and elongation. The degradation study was performed, and the degraded samples were assessed by following pH change and calculating molecular weight reduction to evaluate the the neutralizing effect of Mg(OH)_2_.

## Methods

### Materials

Poly(L-lactide) raw material (PLLA, average Mw = 380 kDa) was supplied by Samyang Co. (Seoul, Korea). Poly(L-lactide-co-*ε*-caprolactone) (PLCL, average Mw = 380 kDa) composed with 50:50 or 75:25 ratio of L-lactide and *ε*-caprolactone units was obtained from DURECT Co. (Birmingham, AL, USA). Chloroform and magnesium hydroxide (Mg(OH)_2_) were purchased by Baker Chemicals Inc. (Center Valley, PA, USA) and Junsei Chemical Co., Ltd. (Tokyo, Japan), respectively. All chemicals were reagent grade and used without further purification.

### Fabrication of PLLA/PLCL/Mg(OH)_2_ blends

The PLLA, PLCL, and Mg(OH)_2_ were mixed by solvent casting method to form a homogenous mixture. First, PLLA pellets were dried in a vacuum oven at 60 °C for 24 h to prevent hydrolysis caused by residual moisture and acidic monomer. Dried PLLA raw materials and PLCL (50:50 or 75:25, respectively) additives were dissolved in 80 mL of chloroform with different rations of PLLA and PLCL, which the total amount of solutes was 5 g in the solution. For Mg(OH)_2_ incorporated samples, 0.25 g of Mg(OH)_2_ powders were added into the above polymer solutions. The mixture was poured into the Teflon mold (100 × 90 × 15 mm^3^), and then the solvent was slowly evaporated at room temperature for 24 h. The film samples were dried under vacuum at 60 °C to remove the residual solvent. The homogeneously mixed composites based on PLLA were chopped up into small fragments under 5 mm, and then kept at room temperature *in vacuo* until thermal process. Pure PLLA fragments were also prepared using the same method. Table 1 lists the abbreviation and conditions of samples including raw PLLA, processed PLLA, PLLA/PLCL, PLLA/Mg(OH)_2_, and PLLA/PLCL/Mg(OH)_2_ blends.

**Table 1:** Various blend compositions of PLCL and Mg(OH)_2_ in PLLA

Abbreviation	PLLA (wt%)	PLCL (50:50) (wt%)	PLCL (75:25) (wt%)	Mg(OH)_2_ (wt%)
Raw PLLA	100	-	-	-
Processed PLLA	100	-	-	-
PLLA95/PLCL5 (50:50)	95	5	-	-
PLLA90/PLCL10 (50:50)	90	10	-	-
PLLA85/PLCL15 (50:50)	85	15	-	-
PLLA80/PLCL20 (50:50)	80	20	-	-
PLLA95/PLCL5 (75:25)	95	-	5	-
PLLA90/PLCL10 (75:25)	90	-	10	-
PLLA85/PLCL15 (75:25)	85	-	15	-
PLLA80/PLCL20 (75:25)	80	-	20	-
PLLA90/PLCL10/Mg5	90	-	10	5

The PLLA only, PLLA/PLCL, PLLA/Mg(OH)_2_, and PLLA/PLCL/Mg(OH)_2_ composites underwent the thermally melting and hot-pressing process using Carver Press 30-12H (Carver Inc., Wabash, IN, USA). Four grams of polymer mixture put on the hot-press plate, and the plate temperature was then raised to 190 °C. After melting time of 3 min, the pressure increased to about 7 tons and maintained for press time of 6 min. The dimension of compressively molded specimens was 150 × 150 mm^2^ and its thickness was 200 μm. All specimens were stored in vacuum oven.

### Characterizations of PLLA/PLCL/Mg(OH)_2_ blends

To evaluate the effects of PLCL and Mg(OH)_2_ additives on the prevention of the molecular weight (Mw) of PLLA during thermal processing, Mw of processed PLLA and PLLA blends was measured by gel permeation chromatography (GPC). The sample was dissolved in chloroform (HPLC grade) at 3 mg/mL concentration, and then the polymer solutions were filtered with a 0.45 μm PTFE filter to remove dust and contaminants. The GPC measurements were performed on an equipment consisted of a Waters 515 HPLC pump, a Waters 717 plus auto sampler and a Waters 410 refractive index (RI) detector (Waters, USA) with KF 804 L and KF 803 (7 μm, 8 × 300 mm, Shodex, Japan) columns (molecular weight resolving range of 500–400,000 Da). The GPC columns were performed using chloroform as the eluent with flow rate of 1 mL/min, and Mw were calibrated with polystyrene standard.

The mechanical properties such as tensile strength and elongation were measured by a universal testing machine (UTM, Instron Co., USA) in accordance with ASTM standard D638. For dynamic tensile test, the processed PLLA and composites with PLLA and additives were prepared with dumbbell shaped specimens (45 × 6 × 2 mm^3^, length × width × thickness) cut by a punch of a compression-molding machine. All test samples were tested after 1 week to remove the internal stress. The specimens were carried out with crosshead speed of 4 mm/min till failure to determine the influence of additives in PLLA on mechanical property.

The thermal properties of processed PLLA and PLLA blends were investigated by differential scanning calorimetry (DSC, TA Instrument, USA) and thermogravimetric analyser (TGA, TA Instruments, USA), respectively. The glass transition temperature (T_g_) of samples was determined by using DSC analysis. The temperature scan was performed with a heating and cooling rate of 10 °C/min under nitrogen atmosphere. The samples were heated from 30 to 210 °C, held for 1 min to erase thermal history effects and cooled to 30 °C, held for 1 min and then heated to 210 °C again for the second scan, which was used to determine the T_g_. The effects of additives types and contents on the thermal stability were assessed through TGA. The melting (T_m_) and crystallization (T_c_) temperatures, in addition to the associated enthalpy of melting (∆H_m_) were measured using a heating/cooling ramp from 20 to 600 °C. A heating rate of 10 °C/min was used under nitrogen atmosphere and at a flow rate of 20 mL/min. Dry sample weighing about 1 mg was used. The standard uncertainty of the sample mass measurement is ±1 %.

### Degradation test

To study the degradation behavior including pH change, mass loss, and molecular weight reduction of PLLA and PLLA blends, four groups (PLLA, PLLA/PLCL, PLLA/Mg(OH)_2_, PLLA/PLCL/Mg(OH)_2_) were prepared in replicate experiments (*n* = 20, each). Pre-weighted specimens (*W*_*0*_) were immersed into 5 mL of phosphate-buffered saline (PBS, pH 7.4) solution at 60 °C under accelerated weathering conditions [21, 22]. The pH of the PBS solution was monitored every 2 days by a pH meter (Hanna Instrument, USA). Temperature and pH of the PBS solution were monitored during the degradation period and they remained at 37 °C and 7.4, respectively.

The pH change, was measured at the predetermined time point (1, 3, 5, and 7 days) to confirm acidity of samples as well as the neutralization of lactic acids with Mg(OH)_2_. At time intervals, the specimens were accurately weighed after deionized water rinse and vacuum drying for 48 h (*W*_*t*_) to determine the mass loss and the reduction of molecular weight. The residual weight percentage was calculated according to the following equation: Mass loss (%) = (*W*_*0*_ − *W*_*t*_)/*W*_*0*_ × 100 [23]. The reduced molecular weight of degraded specimens (*M*_*t*_) was estimated using GPC and compared with an initial molecular weight of samples (*M*_*0*_). The reduction of molecular weight (%) was also calculated according to the following equation: Mw reduction (%) = (*M*_*0*_ − *M*_*t*_)/*M*_*0*_ × 100.

### Statistical analysis

The data were presented as mean ± standard deviation (SD). The results obtained by ANOVA were carried out using origin programs. The significance level considered was 0.05 and data groups were different from others.

## Results and Discussion

### PLLA/PLCL blends

**Fig. 2:**
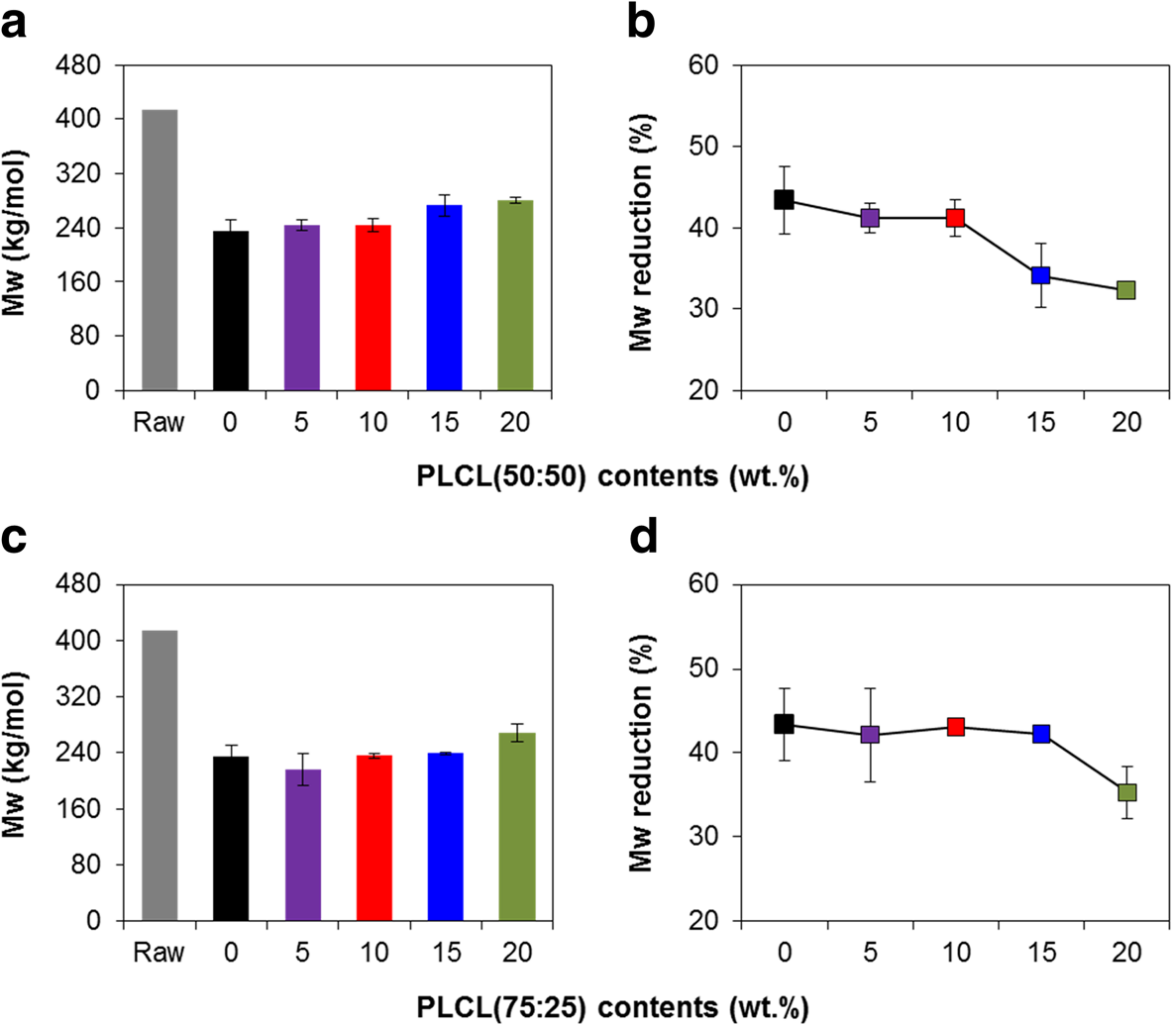
Molecular weight properties of PLLA blends with various **a, b** PLCL (50:50) and **c, d** PLCL (75:25) contents

The molecular weight and reduction degree of thermally processed PLLA and PLLA/PLCL blends with different feed ratios were shown in Fig. 2. The molecular weight of processed PLLA rapidly decreased to 234,342 Da as compared with raw PLLA (414,256 Da), which is mainly caused by random scission to generate acidic oligomers and monomers under thermal processing [6]. These oligomers and monomers with carboxyl and hydroxyl end groups as nucleophiles could attack ester linkages on PLLA backbone *via* intermolecular transesterification and backbiting, and the decomposition of PLLA matrix was accelerated by increasing acidic byproducts and esterification reaction [24]. In contrast, the addition of PLCL (50:50 or 75:25) with increasing PLCL content (5–20 wt%) into PLLA matrix slightly increased the molecular weight of PLLA blends after thermal processing compared with processed PLLA (Fig. 2a and c). With reflecting these results, the reduction degree of molecular weight gradually decreased with increase in PLCL ratio of PLLA/PLCL blends (Fig. 2b and d). Especially, PLCL (50:50) was more effective than PLCL (75:25) on inhibition of molecular weight reduction. The increased proportion of *ε*-caprolactone in polymer blends tended to reduce the thermal degradation due to greater thermal stability of *ε*-caprolactone than that of lactic acid [25]. That is why aliphatic polycaprolactone block in PLCL has one ester linkage with hexanoate repeat unit, whereas polyester PLLA has two ester linkages within repeat unit. This result supposed that the thermal decomposition and formation of acidic byproducts of PLLA/PLCL blends occurred much less than only PLLA at high temperature, and the molecular weight reduction of PLLA/PLCL blends was consequently prevented.

**Fig. 3:**
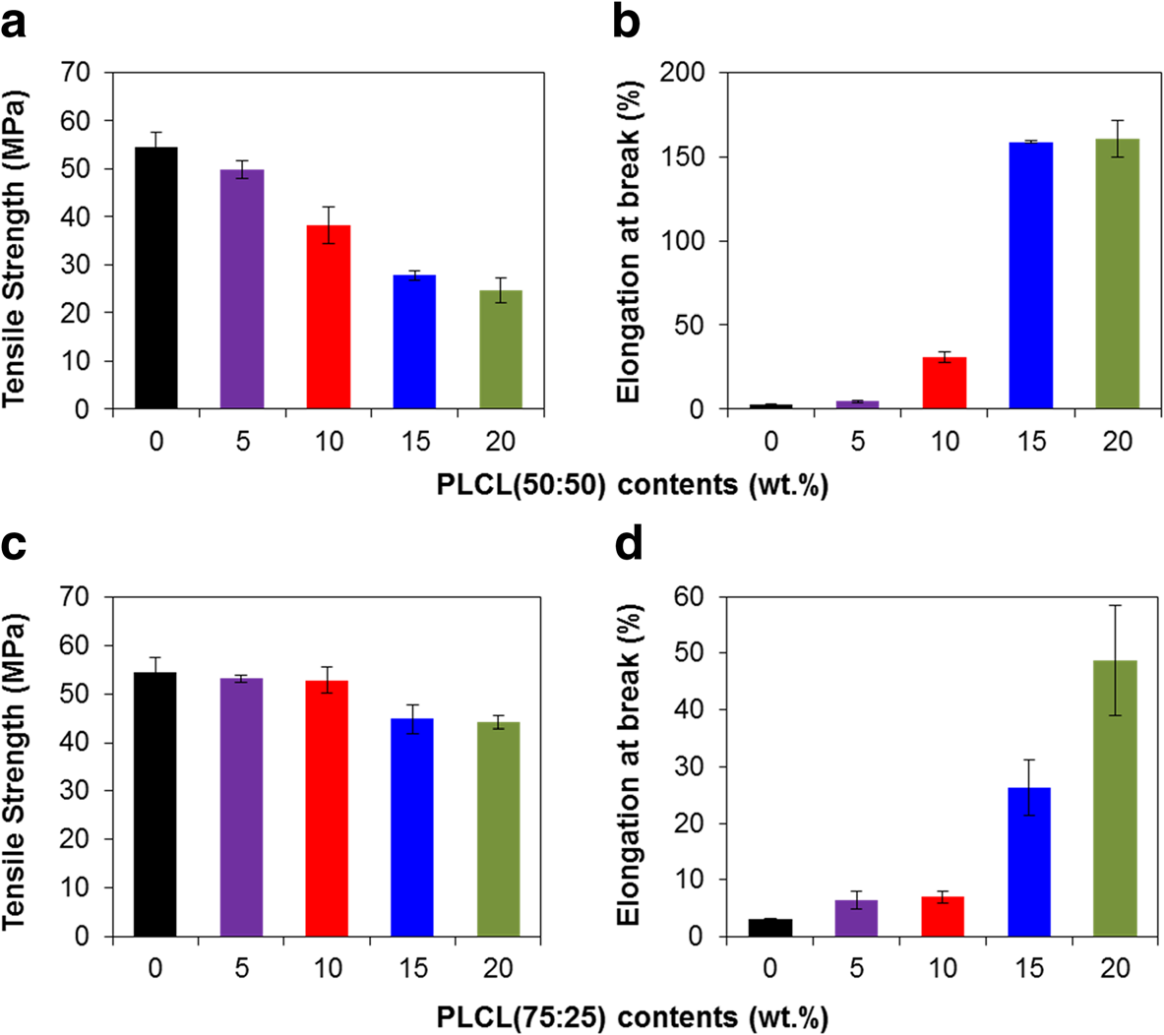
Mechanical properties of PLLA blends with various **a, b** PLCL (50:50) and **c, d** PLCL (75:25) contents

The mechanical properties such as tensile strength and elongation of PLLA/PLCL blends with different PLCL contents were evaluated as compared with thermally processed PLLA as a control. Figure 3 showed that the processed PLLA has higher tensile strength than PLLA/PLCL blends, while the elongation at break of the processed PLLA is severely low compared with PLLA/PLCL blends. It also is observed that the tensile strength decreased, whereas elongation dramatically increased with increasing proportion of PLCL (50:50 or 75:25) in PLLA/PLCL blends. The PLLA exhibited tensile strength of 54.4 MPa. The tensile strength decreased to 24.6 and 44.2 MPa by blending 20 wt% of PLCL (50:50) and PLCL (75:25) in PLLA, respectively (Fig. 3a and c). The elongation of PLLA/PLCL (50:50) blends with different ratios in 95:5, 90:10, 85:15, and 80:20 was 4.8, 31.1, 158.7, and 160.4 %, respectively (Fig. 3b). In PLLA/PLCL (75:25) blends, the elongation increased from 6.4 to 48.8 % with increasing PLCL contents from 5 to 20 %, compared with PLLA (3.0 %) (Fig. 3d). The result demonstrated that PLCL as a plasticizer could improve the flexibility of PLLA but remarkably reduce the tensile strength. In particular, the tensile strength of PLLA/PLCL (50:50) was thoroughly reduced, which could be limited to the application being considered. On the other hands, PLCL (75:25) blended PLLA relatively exhibited similar tensile strength to only PLLA with high flexibility. It is well known that polycaprolactone is highly amorphous, imparting flexibility comparable to a semi-crystalline PLLA. In PLLA and PLCL blended matrix, the matrix became flexible with increasing proportion of polycaprolactone. The ratio between semi-crystalline PLLA and amorphous polycaprolactone is important for proper mechanical property, because the amorphous structure makes the matrix not only flexible but also weak. PLCL (50:50) is composed of twice more caprolactone content than that of PLCL (75:25). Therefore, the tensile strength of PLLA blending with PLCL (50:50) was lower than that of PLLA blending with PLCL (75:25). In mechanical properties results, the PLLA90/PLCL10 (50:50 and 75:25) exhibited to successfully prevent the molecular weight reduction and to properly enhance the flexibility of PLLA.

**Fig. 4:**
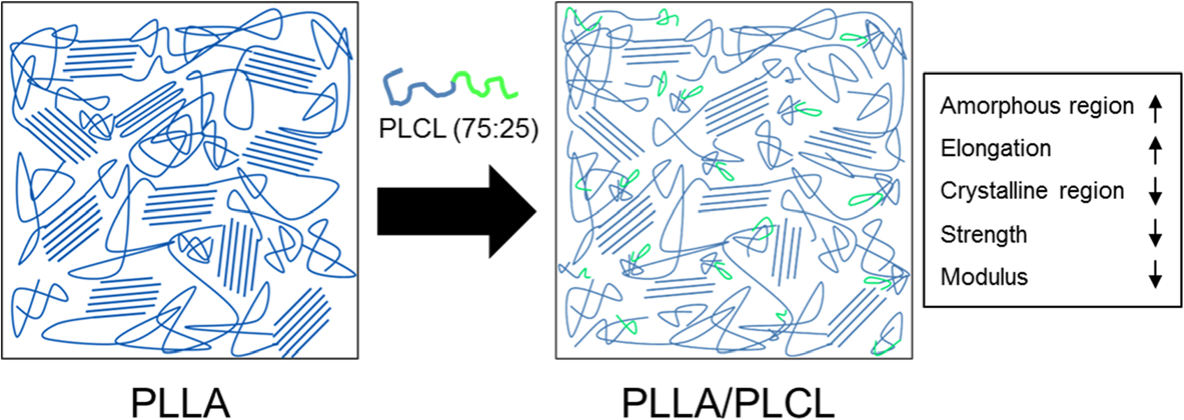
Illustration of the preparation of PLLA/PLCL (75:25) composite film with improved processibility

The thermal analysis of PLLA/PLCL blends was identified by DSC and confirmed by TGA. Table 2 summarized the thermal characteristics including the glass transition temperature (T_g_), melting temperature (T_m_), and melting enthalpy (ΔH_m_) of processed PLLA and PLLA/PLCL blends, acquired from DSC in second heating scan. The results indicated that T_g_ and ΔH_m_ of PLLA/PLCL (50:50 or 75:25) blends decreased with increasing PLCL content, an endothermic melting peak indicated around 177 °C. The values of T_g_ and ΔH_m_ of PLLA were measured to be 61 °C and 52 J/g, respectively. T_g_ value not only decreased by adding PLCL but also gradually decreased with increasing PLCL content. Moreover, the value of ΔH_m_ decreased from 52 to 28 and 34 J/g for PLCL (50:50) and PLCL (75:25) corresponding to PLCL content from 0 to 20 wt%, which clearly indicated the reduced PLLA crystallization with PLCL. Moreover, T_m_ value of PLLA slightly decreased with the addition of PLCL. As described above, the thermal properties such as T_g_, ΔH_m_ and T_m_ strongly influenced by polymer molecular structure [26]. In general, decreasing the crystalline of a semi-crystalline polymer is accompanied by decrease in modulus, stiffness, yield stress, melting point, T_g_, and dimensional stability, and by increase in impact resistance, elongation, and thermal expansion [27]. These results in semi-crystalline PLLA/PLCL blends could be attributed to the decreasing crystalline of PLLA with addition of PLCL containing amorphous polycaprolactone black, as shown in Fig. 4. Although the crystalline domain imparted much mechanical strength and stability to the PLLA/PLCL matrix, the amorphous structure absorbs a substantial fraction of the applied strain energy, owing to its higher elastic compliance [28].

**Table 2:** Thermal properties of PLLA control and PLLA/PLCL blends

Material	T_g_ (°C)	T_m_ (°C)	ΔH_m_ (J/g)
Raw PLLA	59	176	49
Processed PLLA	61	178	52
PLLA95/PLCL5 (50:50)	58	177	41
PLLA90/PLCL10 (50:50)	56	177	36
PLLA85/PLCL15 (50:50)	55	175	32
PLLA80/PLCL20 (50:50)	52	174	28
PLLA95/PLCL5 (75:25)	58	177	54
PLLA90/PLCL10 (75:25)	57	177	46
PLLA85/PLCL15 (75:25)	55	177	38
PLLA80/PLCL20 (75:25)	55	176	34

T_g_: glass transition temperature; T_m_: melt temperature; Δ H_m_: heat of melting

**Fig. 5:**
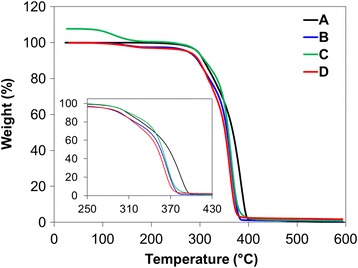
The TGA thermograms of processed PLLA and PLLA blends: **a** Raw PLLA, **b** Processed PLLA, **c** PLLA90/PLCL10 (50:50), and **d** PLLA90/PLCL10 (75:25) blends

TGA was also carried out on the different formulations to determine the effect of PLCL 10 wt% (50:50 or 75:25) on the thermal stability of PLLA (Fig. 5). It revealed that the decomposition temperature of PLLA90/PLCL10 blends was slightly high compared to the processed PLLA control. The addition of PLCL (50:50 or 75:25) affected the thermal stability of PLLA at high temperature ranges over 290 °C. The thermal degradation of PLLA90/PLCL10 blends occurred through ester pyrolysis reaction and induced chain cleavages to be randomly distributed [29, 30]. Since the proton in the CH group of the main chain of PLLA is labile, it has been suggested that the proximity of this labile proton to the ester group affects the thermal sensitivity of the polymer. Thus, the PLLA blending with PLCL with one ester linkage per one repeating unit was more thermally stable than only PLLA with two ester linkage per one repeating unit. In addition, PLLA90/PLCL10 (50:50) has a relatively high thermal stability as compared with others due to the lower proportion of polylactide in whole matrix. Although flexibility and thermal properties of PLLA blending with PLCL (50:50) were better than that of PLLA/PLCL (75:25), its mechanical strength was severely poor for applying to a polymeric biomaterial in medical applications. In this regard, further study on PLLA/PLCL/Mg(OH)_2_ matrix will be carried out by using PLLA90/PLCL10 blends with PLCL ratio of 75:25 due to its proper mechanical property.

### PLLA/PLCL/Mg(OH)_2_ blends

**Fig. 6:**
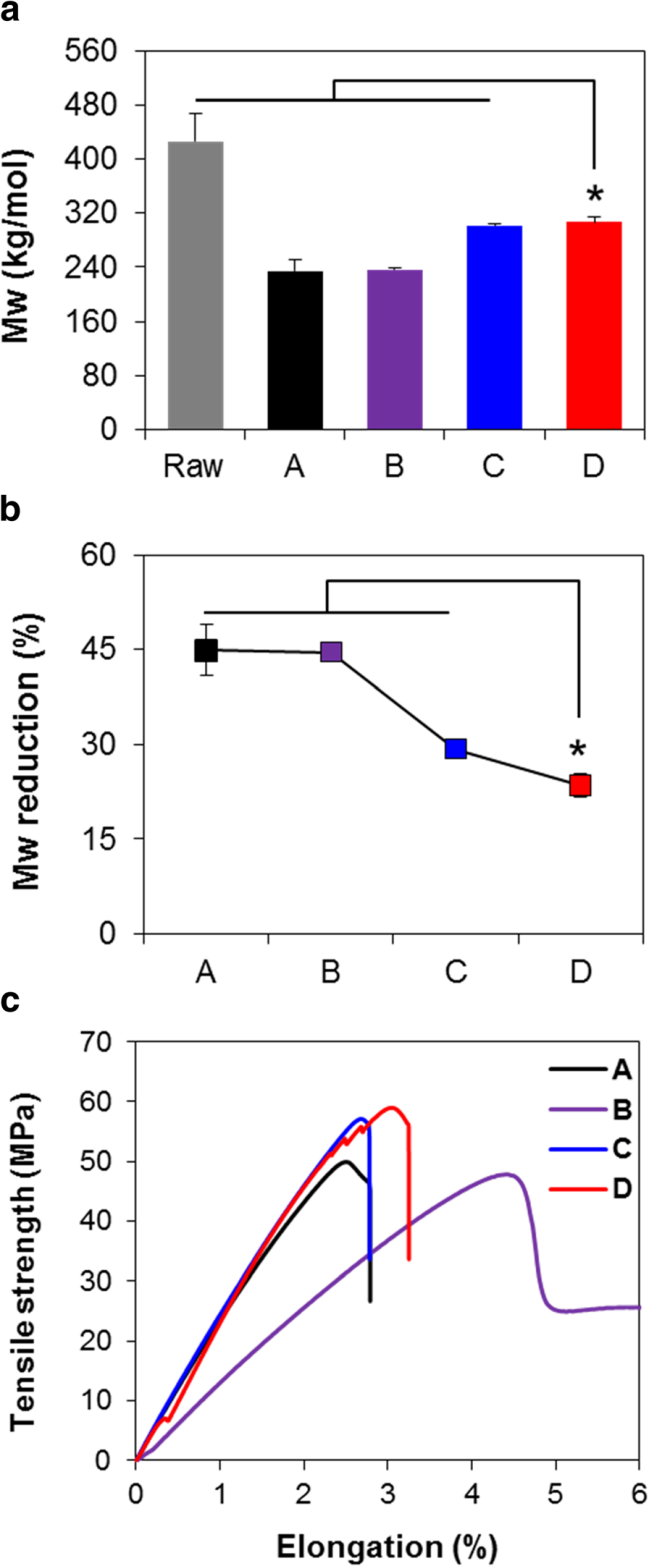
Effects of the additives on PLLA based composite films on **a**, **b** molecular weight and **c** tensile properties: A) processed PLLA, B) PLLA90/PLCL10, C) PLLA100/Mg5, and D) PLLA90/PLCL10/Mg5 blends (^*^ *p* < 0.05)

The PLCL (75:25) and Mg(OH)_2_ (coded to Mg) blended PLLA samples were fabricated by thermal processing. PLLA90/PLCL10/Mg5 composite was characterized and compared with thermally processed PLLA, PLLA90/PLCL10, and PLLA100/Mg5 samples. Figure 6a and b were observed that the thermal decomposition of PLLA was alleviated by adding Mg(OH)_2_ as well as PLCL. The molecular weight of PLLA100/Mg5 and PLLA90/PLCL10/Mg5 increased compared with processed PLLA, and thus the reduction degree greatly decreased, which indicated that Mg(OH)_2_ improved the thermal stability. Mg(OH)_2_ as a basic inorganic compound degraded as [Mg^2+^] and [OH^−^]. One Mg^2+^ of Mg(OH)_2_ also needs two anionic compounds to neutralize the charge and thus binds to anionic terminal group of PLLA and/or acidic degradation product; [Mg^2+^] · 2[lactic acid–OH^−^]. The [Mg^2+^] · 2[lactic acid–OH^−^] form inactivates hydroxyl groups of PLLA and byproducts which are capable of backbiting reaction and hydrolysis of ester linkages incorporated into the polymer backbone [31–34]. It seemed that the Mg(OH)_2_ in PLLA90/PLCL10/Mg5 matrix inhibited consecutive ester pyrolysis and thermal-hydrolysis reactions with binding anionic terminal group of polymer and acidic byproducts generated during thermal processing. However, the conclusive mechanism was not fully understood yet. The inorganic Mg(OH)_2_ particles slightly enhanced the tensile strength affected by molecular weight, but reduced the elongation at break due to poor interfacial adhesion, as shown in Fig. 6c. PLLA100/Mg5 matrix exhibited higher tensile strength than PLLA but still brittle fracture characteristics like PLLA. In PLLA90/PLCL10/Mg5 matrix, the tensile strength and flexibility appropriately increased due to Mg(OH)_2_ and PLCL, respectively, compared with PLLA control. Mg(OH)_2_ not only affected the molecular weight reduction and mechanical strength under thermal processing, but also alleviated reduced pH value and molecular weight during degradation.

**Fig. 7:**
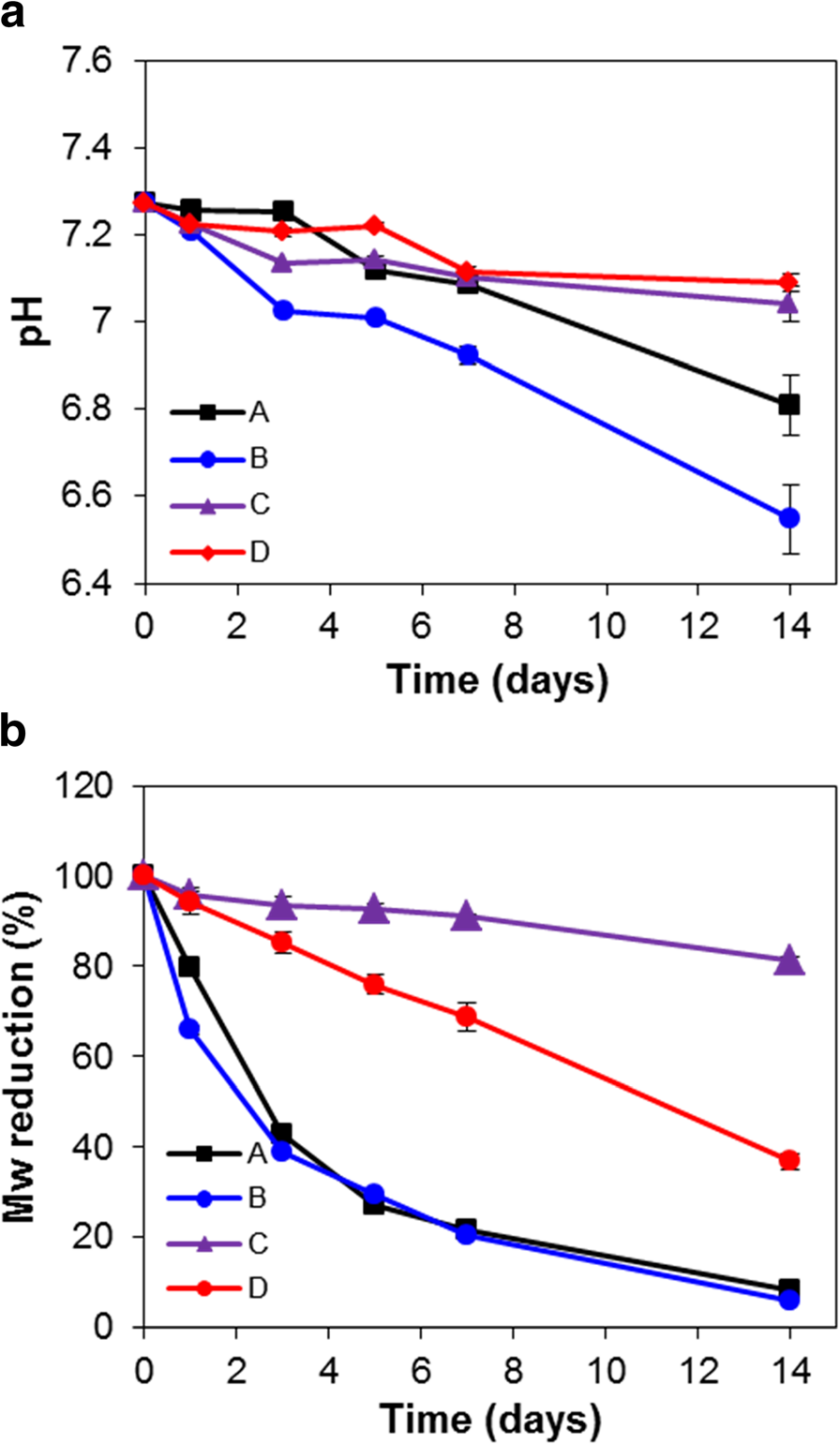
Degradation study of **a** changes of pH and **b** reduction of molecular weight with hydrolytic time: A) processed PLLA, B) PLLA90/PLCL10, C) PLLA100/Mg5, and D) PLLA90/PLCL10/Mg5 blends

Figure 7 displayed the results in accelerated degradation of PLLA, PLLA100/Mg5, PLLA90/PLCL10, and PLLA90/PLCL10/Mg5 blends in PBS (pH 7.4) at 60 °C for 14 days. As shown Fig. 7a, the pH values of media containing PLLA and PLLA90/PLCL10 samples decreased to 6.8 and 6.5, respectively because of the acidic byproducts of PLLA and PLCL. The lower pH value of the PLLA/PLCL degraded media depended on caprolactone segment, which was easily degenerated due to T_g_ value below 60 °C. Meanwhile, the Mg(OH)_2_-containing matrix such as PLLA100/Mg5 and PLLA90/PLCL10/Mg5 slightly compensated the pH as compared with PLLA control, corresponding to 7.1 in 14 days. For pH neutralization of the degradation medium, many researchers studied some inorganic compounds such as sodium bicarbonate and calcium carbonate and incorporated these inorganic compounds into the PLLA matrix to evaluate their effect on the degradation process [10, 35]. In particular, Mg(OH)_2_ particles can neutralize the acidic environment since the dehydrated magnesium ion can bind with two moles of anionic compounds [19]. The acidic degradation products of degraded PLLA and PLCL were sufficiently neutralized by Mg(OH)_2_ particles, and also the accelerated formation of degradation byproducts was prevented by binding of acidic terminal groups of PLLA and PLCL to Mg(OH)_2_.

The molecular weights of degraded samples were evaluated to confirm the effects of Mg(OH)_2_ on molecular weight reduction as well as pH balance (Fig. 7b). The PLLA only and PLCL90/PLCL10 blends almost degraded and their molecular weights were dropped to about 10 % for 14 days. On the other hand, the molecular weight of Mg(OH)_2_-containing blends slowly declined, and, in particular, PLLA100/Mg5 scarcely decomposed as compared with other groups due to higher T_g_ value of PLLA than that of PLLA90/PLCL10. During degradation, a large amount of the acidic degradation products were released, which usually resulted in a rapid decrease in the pH of the soaking medium [36]. The acidic byproduct reacted like a nucleophile to ester linkage of PLLA and PLCL backbones and then accelerated the decomposition of polymer chains. In PLLA blends with Mg(OH)_2_, the Mg(OH)_2_ hindered the accumulation of acidic byproducts and averted backbiting and intermolecular transesterification. These results demonstrated that the Mg(OH)_2_ affected the prevention of molecular weight reduction by neutralizing acidic substance.

## Conclusions

The new PLLA blends with PLCL and Mg(OH)_2_ additives successfully fabricated by thermal processing, and the effects of PLCL and Mg(OH)_2_ additives on physico-chemical and thermal properties under thermal decomposition of PLLA matrix were assessed using various analyses. With increasing the amount of PLCL, the PLLA/PLCL blends exhibited the alleviation of molecular weight reduction and the improvement of flexibility compared with thermally processed PLLA control. The PLLA/PLCL blends with increasing proportion of polycaprolactone depicted proper thermal stability as well as mechanical strength including tensile strength and elongation. In Mg(OH)_2_-containing matrix, the molecular reduction and mechanical strength were dramatically improved because the dehydrated magnesium ion inhibited the decomposition of polyester substrate by counteracting acidic compounds, which is a kind of nucleophile at ester linkage. In particular, the Mg(OH)_2_ in matrix certainly neutralized the acidic byproducts involved during polyester degradation, that caused the acid-induced inflammatory reaction *in vivo*. The obtained results suggested that PLCL and Mg(OH)_2_ additives were effective to enhance flexibility and control degradation behavior of biodegradable PLLA matrix, and therefore the PLLA/PLCL/Mg(OH)_2_ composites have the potential as a material for bio-absorbable biomedical devices such as implants and stents.
